# Differential Expression of Signaling Pathway Genes Associated With Aflatoxin Reduction Quantitative Trait Loci in Maize (*Zea mays L.*)

**DOI:** 10.3389/fmicb.2019.02683

**Published:** 2019-11-26

**Authors:** Felicia Parish, W. Paul Williams, Gary L. Windham, Xueyan Shan

**Affiliations:** ^1^Department of Biochemistry, Molecular Biology, Entomology, and Plant Pathology, Mississippi State University, Starkville, MS, United States; ^2^United States Department of Agriculture, Agricultural Research Service, Maize Host Plant Resistance Research Unit, Starkville, MS, United States

**Keywords:** signaling pathway genes, maize, *Aspergillus flavus*, aflatoxin reduction, quantitative trait loci

## Abstract

The roles of signaling pathway genes related to the aflatoxin reduction trait in maize were studied for the improvement of maize resistance to the fungal pathogen *Aspergillus flavus* (*A. flavus*). In this study, 55 maize genes in plant–pathogen interaction signaling pathways were investigated among 12 maize near-isogenic lines (NILs) that carry maize quantitative trait loci (QTL) associated with aflatoxin reduction. These maize NILs were developed from maize inbred lines Mp313E (resistant donor parent) and Va35 (susceptible recurrent parent). The quantitative RT-PCR (qRT-PCR) technique was used to study the gene expression patterns. Seven calcium-dependent protein kinases and one respiratory burst oxidase displayed significant differential expression levels among the maize QTL-NILs. In addition, the gene expression profiles of WRKY transcription factors were also examined. Maize WRKY 52, WRKY 71, and WRKY83 genes displayed significantly differential expression levels among the QTL-NILs. The elucidation of differentially expressed signaling pathway genes involving maize resistance to *A. flavus* can provide insights into maize disease resistance and enhance maize molecular breeding.

## Introduction

*Aspergillus flavus* (*A. flavus*) is a serious pathogen to developing maize ears. *A. flavus* is of particular interest because it is involved with the production of the carcinogenic aflatoxins. Aflatoxins are secondary metabolites of *A. flavus* and the contamination they cause greatly affects the quality and safety of maize products. A variety of health issues are associated with ingestion of aflatoxins with both humans and animals. Aflatoxin B_1_ is a potent liver toxin and carcinogen (Eaton and Gallagher, 1994). The United States Food and Drug Administration established explicit guidelines to prevent hazardous amounts of aflatoxin exposure. However, high temperatures and drought provide favorable conditions for *A. flavus* contamination in maize. Researchers have proposed models estimating monetary losses in the range of $52.1 million to $1.68 billion dollars annually related to aflatoxin contamination of maize grain (Diener et al., 1987; Mitchell et al., 2016). These findings emphasized the need for intervention to reduce aflatoxin contamination in maize production.

The identification of host plant resistance genes and genetic factors associated with aflatoxin reduction is an important strategy to increase effectiveness of maize yield. The elucidation and utilization of maize resistance QTL represent a significant advancement in mitigating this plant pathogen. The maize QTL regions involved in this study have previously been described as contributing to aflatoxin reduction in maize inbred line Mp313E (Brooks et al., 2005; Warburton et al., 2009; Willcox et al., 2013). Twelve maize QTL-NILs were selected by backcrossing the QTL regions at maize chromosome bin locations 2.05, 3.05, 4.06, and 4.09 from maize inbred line Mp313E (resistant) into maize inbred line Va35 (susceptible). These breeding efforts can be enhanced with knowledge of maize defense mechanisms and genes in signal transduction pathways. In plants, the first steps in molecular responses of a pathogenic attack are to perceive the pathogen patterns and turn on the signaling pathways. These signaling pathways eventually lead to the expression of defense genes and the inhibition of pathogenic processes. Several signaling pathways have been characterized to initiate immune responses in plant when under attack by pathogens. These signaling pathway components are mainly protein kinases and transcription factors. Analysis of the relationships of plant signaling pathways with the resistance QTL regions present in the maize QTL-NILs will provide insights into the underlying mechanisms of maize resistance to *A. flavus*.

The plant immune responses consist of complex, cell-mediated defense patterns against a multitude of invading pathogens. Fungi such as *A. flavus* have the characteristic ability to invade plant epidermal cells and surround the exterior of the cells with hyphae (Jones and Dangl, 2006). The downstream signal transduction triggered by the detection of the fungus is important to maize resistance. Signaling pathways are initiated upon interaction of a transmembrane pattern recognition receptor (PRR) with a fungal pathogen-associated molecular pattern (PAMP) (Newman et al., 2013). PAMPs can be defined as conserved, specific patterns located on the pathogen that elicit a specific response from the appropriate PRR interaction on the host. Plants utilize PRRs present on the plasma membrane to detect PAPMs. PAMP detections by these receptors trigger the basal resistance response called pathogen-triggered immunity (PTI) (Cui et al., 2015). However, pathogens have developed the ability to counteract the basal PTI response by releasing effectors and thereby weakening the effects displayed by this initial response. Plants have developed a vigorous response to the pathogen effectors produced as a result of the initial basal response. This wave of robust defense has been termed the effector-triggered immunity (ETI). ETI is deployed by the plant when pathogenic effectors are recognized by pathogenic related proteins (PR proteins) characterized by nucleotide-binding and leucine-rich repeat domains (NB-LRR). These proteins potentiate the second branch of the plant immune system (Jones and Dangl, 2006). The effector response provides an amplification of the original PTI response and induces the hypersensitive response.

The genes involved in the fungal PAMP recognition and other signal transduction pathways have been characterized from a number of plant species and curated in major databases such as the Kyoto Encyclopedia of Genes and Genomes (Kanehisa and Goto, 2000). The PAMP-triggered immunity pathway includes genes of calcium-dependent protein kinase (CDPK), respiratory burst oxidase (RBO/NADPH), and reactive oxygen species (ROS) production. The DNA and protein sequences of these signaling pathway genes in the KEGG database provide the starting material for conducting a comprehensive genome-wide data mining for the corresponding maize genes in the maize genome database MaizeGDB. The objectives of this study were to conduct a genome-wide survey of maize genes involving maize and fungus interaction and signaling pathways and investigate the gene expression levels among the 12 maize QTL-NILs that carry maize resistance QTL regions.

## Materials and Methods

### Plant Materials and Aflatoxin Test

The maize QTL regions were designated as QTL1 (bin 2.05), QTL2 (bin 3.05), QTL3 (bin 4.06), and QTL4 (bin 4.09), respectively. The 12 maize QTL-NILs were the advanced generations originally generated from backcrossing the maize inbred line Mp313E (resistant) into the Va35 (susceptible) background. Each QTL-NIL genotype carries either a single QTL region (e.g., NIL-QTL4) or multiple QTL regions in the genome (e.g., NIL-QTL1,2,3), in the latter case the QTL-NILs were developed by introducing two to three resistance QTL regions from Mp313E into Va35 genome. The 12 maize QTL-NILs as well as the two parental maize lines were planted at R. R. Plant Science Research Center at Mississippi State University, MS. The ears were inoculated 14 days after hand-pollination with the *A. flavus* strain NRRL3357 (ATCC # 200026; SRRC 167) utilizing the side-needle technique described by Windham et al. (2003). The field experimental design was a randomized complete block with split plot and three replications for each genotype. The developing kernel samples were collected 7 days after the inoculation and ground to a powder with liquid nitrogen (N_2_) for future analysis. The remaining primary ears were harvested at maturity and measured for aflatoxin levels. Aflatoxin concentration was determined using the Vicam AflaTest method. Values for aflatoxin concentration were transformed [ln(y + 1)] before statistical analysis. Tests of significance were performed before converting values to geometric means expressed in the original units of measure.

### RNA Extraction

Total maize RNA was extracted from the ground kernel samples with the Aurum^TM^ Total RNA Fatty and Fibrous Tissue Kit from Bio-Rad (Bio-Rad Laboratories, Hercules, CA, United States). The extraction followed the manufacturer’s protocol with the following minor modifications. The ground kernel samples were placed into sterile 2 mL tubes with 100 mg per sample. 1 mL of cold Trizol was added to each sample and vigorously vortexed to ensure a complete suspension of kernel powder. The resulting lysate was incubated at room temperature for 5 min. The tubes containing the lysate were centrifuged at 12,000 rpm for 5 min at 4°C. The Aurum^TM^ Total RNA Fatty and Fibrous Tissue Pack instructions were followed from this point forward.

### cDNA Synthesis

cDNA synthesis was conducted with the Invitrogen Thermoscript^TM^ RT-PCR System (Invitrogen Life Technologies, Carlsbad, CA, United States) adhering to the manufacturer protocol. A total of 10 μL from each sample of previously extracted total RNA was used for the cDNA synthesis. The RNA was denatured at 65°C for 5 min then kept at 4°C. The cDNA synthesis was conducted at 50°C for 60 min, followed by 85°C for 5 min. The synthesized cDNA samples were stored at −20°C until further qRT-PCR analysis.

### Data Mining, Primer Design, and Primer Efficiency

The DNA sequences of genes in the plant pathogen interaction pathways were obtained from the KEGG Pathway Database^1^. More specifically, the DNA sequences of the Plant-Pathogen Interactions 5.10 pathway genes from KEGG were used to blast the MaizeGDB database and search for all potential orthologous maize gene sequences present in the maize (*Zea mays L.*) genome. Some of these genes were found to locate within the resistance QTL regions. All primers were designed with software Primer3. The PCR amplification efficiencies for the designed primers were analyzed before the gene expression analysis. The PCR amplification efficiencies were determined with a standard curve analysis technique. The sample dilution for the standard curve analysis utilized a 5-stage 3-fold dilution series and the mean ΔCp values vs. log of the dilution factors were plotted. The efficiencies of primers were calculated applying the equation Log_2_(*r* + 1), where *r* is the coefficient of determination of the linear standard curve equation. Only primers with efficiency above 0.9 were used for further gene expression analysis. Glyceraldehyde 3-phosphate dehydrogenase (GAPDH) was used as the control housekeeping gene for gene expression data normalization.

### Quantitative Real-Time PCR

Quantitative real-time PCR was performed with Roche Light-Cycler 480 (Roche Diagnostics Operations, Indianapolis, IN, United States). The Roche Light-Cycler 480 SYBR Green 1 Master kit was used for the qRT-PCR analysis. A total of 45 inoculated maize samples (with *A. flavus* strain 3357) were analyzed for gene expression, including 12 QTL-NILs, the two parental inbred lines, and one F_1_ hybrid of Mp313E and Va35. The qRT-PCR program was: (1) 1 cycle of 95°C for 5 min; (2) 45 cycles of 95°C for 10 s, 60°C for 15 s, 72°C for 15 s; (3) 1 cycle of 95°C for 5 s, 65°C for 1 min, 97°C at continuous; (4) 1 cycle of 40°C for 10 s.

### Data Analysis

Phylogenetic trees were constructed with the program MUSCLE and displayed with program MEGA7. The R statistical programing language was used to perform analysis of variance (ANOVA). The significance level was determined at *p* < 0.05. Pearson’s coefficients were calculated on the gene expression data between all pairs of genes for the correlation analysis. The R package “Corrgram” was used to display the Pearson’s coefficients. Network analysis was performed following the manual of the R packages “sna” and “network.”

## Results

### Aflatoxin Reduction Levels in the Selected Maize QTL-NILs

The presence of QTL regions in the 12 maize QTL-NILs were confirmed by genotyping the presence of the DNA markers flanking the QTL1, QTL2, QTL3, and QTL4 chromosome bin regions. To quantify the effects of the resistance QTLs on aflatoxin reduction, the primary ears from the QTL-NILs as well as the two parental maize lines were artificially inoculated, collected at maturity, and measured for aflatoxin concentration. Table 1 shows the average aflatoxin levels (ng/g) determined on per 50 g ground mature kernels for each QTL-NIL with three replications. Generally speaking, greater effects in aflatoxin reduction were observed from QTL-NILs carrying multiple QTLs in each line than those carrying a single QTL in each line. However, exceptions from the above observation were also revealed, indicating interactions exist between genes and QTLs. The parental inbred line Mp313E (resistant genotype) exhibited lowest level of aflatoxin at 11 ng/g, whereas, the parental inbred line Va35 (susceptible genotype and the recurrent parent) exhibited highest aflatoxin level of 805 ng/g. The 12 QTL-NILs exhibited aflatoxin levels between the parental lines (Table 1). Since the aflatoxin reduction effects were resulted from the introduction of the QTL regions from Mp313E to Va35, the QTL-NILs were validated suitable and valuable materials for resistance gene studies.

**TABLE 1:** Mean aflatoxin concentrations measured in the mature kernels for maize QTL-NILs and inbred parents.

**Genotype**	**ln(y+1)**	**ng/g**
Va35 (Susceptible)	6.69	805
Mp313E (Resistant)	3.08	21
NIL-QTL #2, #3, #4	6.13	456
NIL-QTL #1, #2, #3	4.51	90
NIL-QTL #2	5.04	154
NIL-QTL #2, #3	5.66	287
NIL-QTL #1, #2	3.73	41
NIL-QTL #1, #2, #4	5.01	150
NIL-QTL #4	6.62	750
NIL-QTL #1	5.56	284
NIL-QTL #1, #4	5.77	318
Mp313E x Va35	3.7	39
NIL-QTL #3, #4	5.13	168
NIL-QTL #3	5.06	157

*LSD = 1.34.*

### Data Mining of Candidate Maize–Fungus Interaction Pathway Genes

**FIGURE 1:**
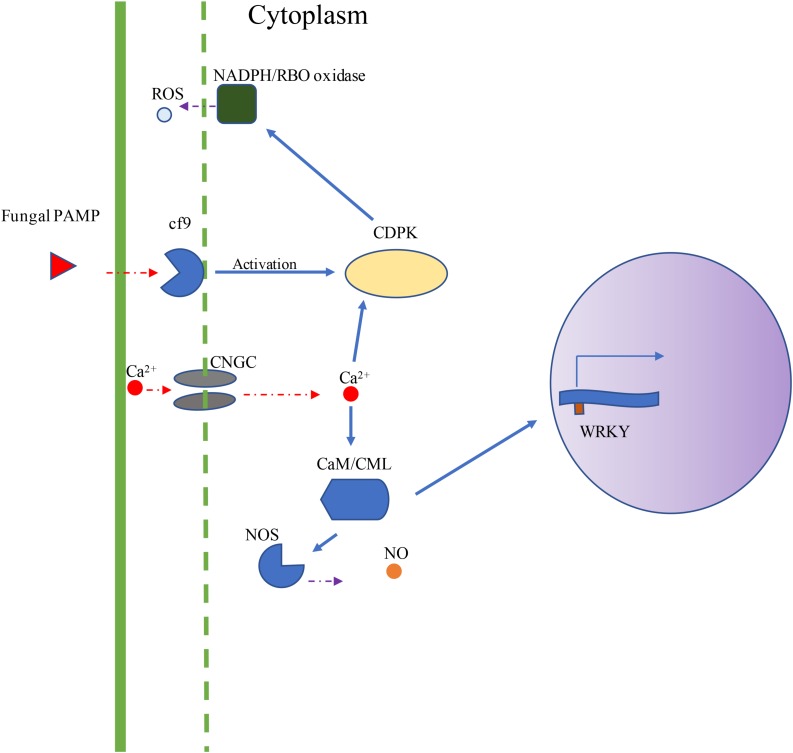
Visual depiction of KEGG Plant–Pathogen Interaction Pathways. The visual depiction summarizes the major signaling pathways obtained from KEGG representing the plant cell interaction with fungal pathogen. Two major pathways were studied for gene expression analysis. The first pathway is the PAMP-triggered immunity pathway (Cf9, CDPK, RBO, and ROS). The second pathway involves CDPKs, CaM/CMLs, CNGCs, and WRKY transcription factors. In this study, maize genes were selected from CDPK, RBO, CaM/CML, CNGC, and WRKY gene families.

Using DNA sequences and protein sequences of genes in the Plant-Pathogen Interaction Pathway 5.10 from KEGG to blast the MaizeGDB database, 117 potential orthologous maize genes were identified in the maize (*Zea mays*) genome (Supplementary Table 1). Figure 1 depicts the plant–pathogen interaction signaling pathways as well as the possible crosstalk that exist between these pathways. Two major pathways were focused on for selection of genes for expression analysis. The first pathway is the PAMP-triggered immunity pathway which involves CDPK, respiratory burst oxidase (RBO), and ROS production. The second pathway involves a calcium related signaling pathway which includes CDPK, calmodulin/calmodulin-like proteins (CaM/CML), cyclic nucleotide-gated channels (CNGC), and WRKY transcription factors (Figure 1). Data mining of maize pathway genes resulted in 35 CDPK genes, 59 CaM/CML genes, 11 RBO genes and 12 WRKY genes for further analysis (Supplementary Table 1).

### Phylogenetic Analysis

**FIGURE 2:**
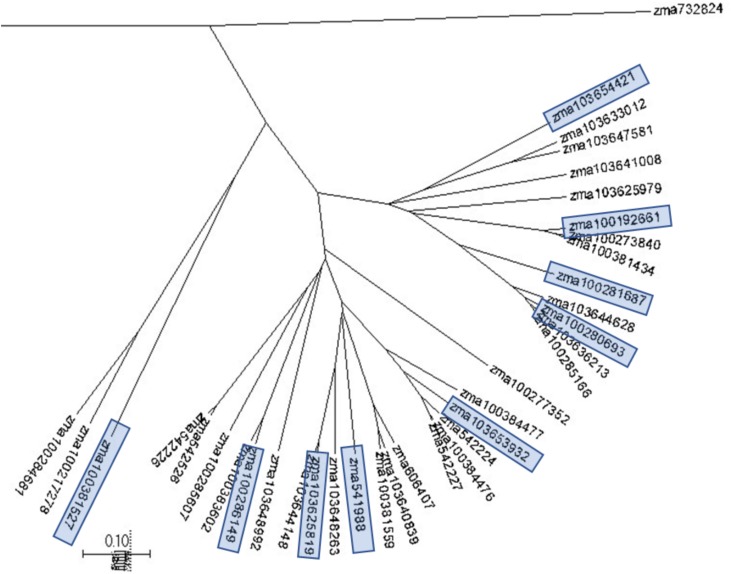
A phylogenetic tree for maize calcium-dependent protein kinases (CDPKs). A phylogenetic tree were constructed to assess the genetic similarities and relationship in maize CDPKs using MUSCLE and displayed with MEGA7. Five major clades were observed from 35 CDPK genes. CDPK genes selected for the subsequent gene expression analysis are highlighted and represented the major clades.

Phylogenetic trees were constructed to assess the genetic similarities and relationship in the selected maize pathway genes with the program MUSCLE and the phylogenetic trees were displayed with program MEGA7. Phylogenetic analysis was conducted through examination of the phylogenic trees by gene families, which are associated with the plant pathogen interaction pathways. Figure 2 displays a phylogenetic tree constructed for the CDPKs. Five major groups were observed from 35 CDPK genes. The selected CDPK genes for the subsequent gene expression analysis represented the major groups. Therefore, each major group in the phylogenetic tree was represented by at least one CDPK gene (Figure 2). Phylogenetic trees were constructed for all gene families to select the genes for primer design and qRT-PCR gene expression analysis.

### Gene Expression Analysis

A total of 55 genes from four gene families were selected as pathway representing genes for the primer design and further qRT-PCR gene expression analysis (Supplementary Tables 1, 2). The PCR efficiency for each pair of gene primers was determined (Supplementary Tables 1, 2). Gene primers that showed efficiencies above 0.9 were selected for further qRT-PCR analysis. The housekeeping gene glyceraldehyde 3-phosphate dehydrogenase (GAPDH) had a PCR efficiency of 0.98. Out of the 55 qRT-PCR gene primer evaluation assays, 20 gene primers had a PCR efficiency > 0.9 (Supplementary Tables 1, 2) and were used in the subsequent qRT-PCR gene expression analysis. Quantitative RT-PCR gene expression analysis revealed a total of 11 differentially expressed genes in calcium related signaling pathways based on *p*-value at significant level of 0.05, including CDPK, CNGC, and WRKY transcription factors (Table 2). No significant deferentially expressed genes were identified in CaM/CML (Table 2).

**TABLE 2:** Differentially expressed signaling pathway genes identified in maize QTL-NILs.

	**KEGG**	**Maize GDB**	***p*-Value**
CDPK	zma103653932	GRMZM2G081310	0.03
	zma103654421	GRMZM2G2332660	0.02
	zma100192661	GRMZM2G311220	0.05
	zma100281687	GRMZM2G104125	0.002
	zma541988	GRMZM2G320506	0.16
	zma100280693	GRMZM2G099425	0.008
	zma100286149	GRMZM2G025387	0.0038
	zma103625819	GRMZM2G027351	0.025
CaM/CaML	zma100285141	GRMZM2G115628	0.17
	zma100282040	GRMZM2G155822	0.1
	zma100193164	GRMZM2G142693	0.09
RBO	zma10010532	GRMZM2G043435	0.0055
WRKY	zma100275623/WRKY52	GRMZM2G151407	0.0017
	zma100383070/WRKY72	GRMZM5G816457	0.14
	zma100384128/WRKY71	GRMZM2G052671	0.01
	WRKY83	GRMZM2G012724	0.05

**FIGURE 3:**
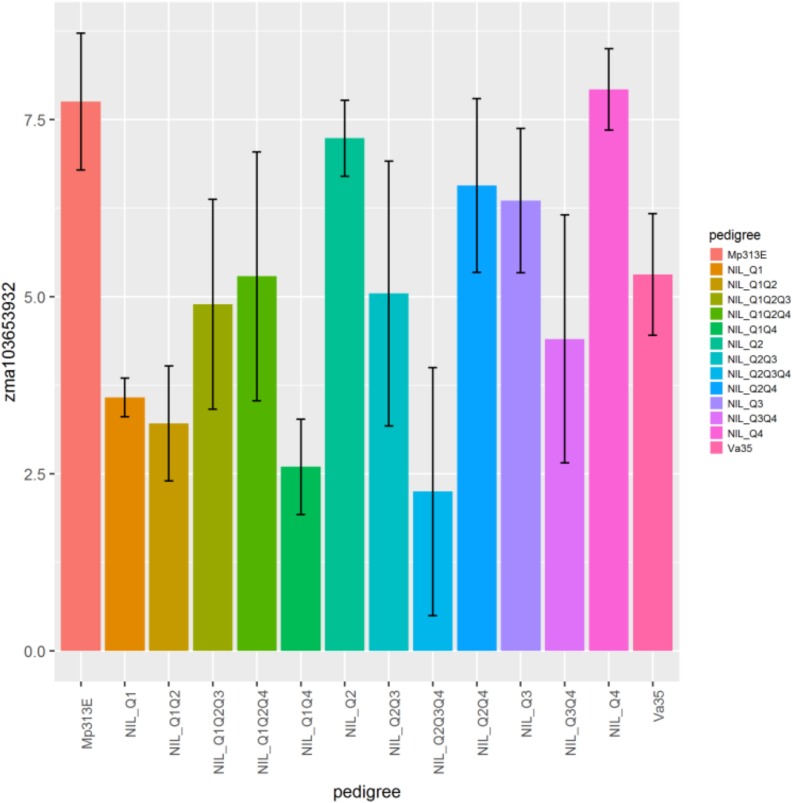
Relative gene expression levels of a calcium-dependent protein kinase gene, zma103653932 (GRMZM2G081310) in maize QTL-NILs. A plot showing the relative expression levels of gene *zma103653932* (*GRMZM2G081310*) in maize QTL-NILs. The relative gene expression levels were presented in terms of the average ΔΔCp values obtained from qRT-PCR analysis. This data represent summarization of ΔΔCp values from samples of a field experimental design that was a randomized complete block with split plot and three replications for each QTL-NIL. The significance level of differential gene expression of the CDPK *zma103653932* gene is determined at *p* < 0.05.

The genes *zma103653932, zma1036544221, zma100192661, zma100281687, zma100280693, zma100286149, zma103625819* displayed the significant differential gene expression (*p* < 0.05) (Table 2). Gene *zma103653932* is a CDPK based on sequence alignment with the maize B73 genome sequences in MaizeGDB. More specifically this gene is categorized as cdpk7. *Zma103653932* is located within the QTL 3 region on chromosome 4. Figure 3 shows the expression levels of *zma103653932* expressed among the QTL-NILs. It is highly expressed in the QTL-NILs carrying single QTL2, QTL3, QTL4, and a combined QTL2 and QTL4. Computational protein structure analysis revealed unique characteristics of *zma103653932* protein such as possessing both a kinase domain and a calmodulin domain in one gene product (data not shown). The calmodulin domain of *zma103653932* protein is found to be highly related to the soybean CDPK regulatory region. Gene *zma100281687* is a second CDPK with significant differential gene expression with a *p*-value of 0.002. Gene *zma100280693* is a differentially expressed CDPK located on chromosome 2. Gene *zma100286149* is a CDPK designated on chromosome 8. The overall trend for all differentially expressed CDPKs was very similar with the highest gene expression showed in the QTL-NILs carrying the single QTL2, QTL3, and QTL4 regions.

One RBO gene, *zma10010532*, was found to be significantly differentially expressed among the QTL-NILs at *p* < 0.05. The gene *zma1001532* encodes a respiratory burst NADPH oxidase family enzyme. Upon further protein sequence analysis, this enzyme contains a ferric reductase-like transmembrane component, a FAD-binding domain (FAD binding 8), and a ferric reductase NAD binding domain (NAD binding 6). Interestingly, this RBO gene showed higher levels of expression in the QTL-NILs carrying the single QTL2, QTL3, and QTL4 regions, similar to the trend observed in CDPKs. The genes of *WRKY 52*, *WRKY 71, WRKY72*, and *WRKY 83* contain the WRKY DNA binding domains. The expression of gene *WRKY72* showed similar patterns to CDPK genes with NIL-QTL2, NIL-QTL4, and NIL-QTL2,4 lines exhibiting higher expression levels. However, the highest expression for *WRKY 52* gene was found in NIL-QTL1,2,3 with a *p*-value of 0.001. *WRKY 83* exhibited a slight difference with the NIL-QTL2 line showing higher gene expression.

### Gene Expression Analysis by Correlogram and Network

**FIGURE 4:**
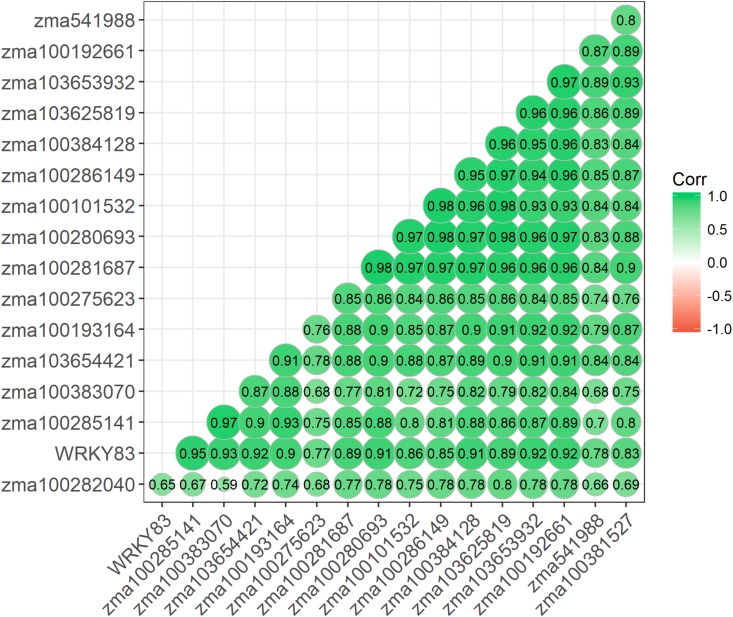
A Correlogram displaying the Pearson’s coefficients on all gene pairs using expression data of the significant candidate genes. Pearson’s coefficients were calculated based on the gene expression data between all pairs of the signaling pathway genes studied. A correlogram is a direct visual display of the matrix of Pearson’s coefficients. By this method, correlations between genes are displayed by grouping genes that have similar expression patterns, and the size of the color-coded circles proportionally represents the values of Pearson’s coefficients. Green color represents positive correlations.

**FIGURE 5:**
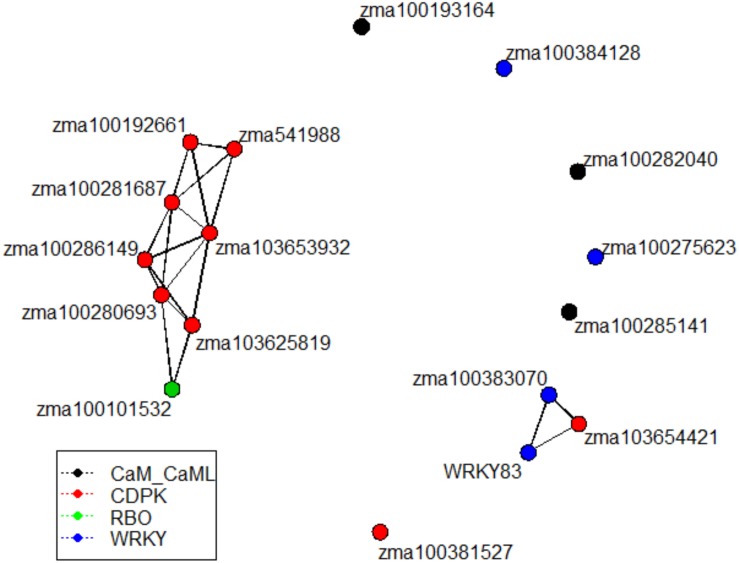
The empirical gene network constructed from the gene expression data. Network was constructed using the Euclidean distance from the first two principal component scores of gene expression data analysis. Network were generated to display the empirical relations among signaling pathway genes. The vertices were color-coded to represent different gene families. The genes connecting in the network were potentially closely interacting genes in the maize fungus interaction signaling pathways.

Given similar gene expression patterns observed among some of the signaling pathway genes, further statistical correlation studies were conducted using the gene expression data. A correlogram was generated to display the matrix of Pearson’s coefficients calculated from the gene expression data between all pairs of the signaling pathway genes (Figure 4). All the significantly expressed signaling pathway genes investigated in this study showed positively correlation based on the gene expression patterns among the maize QTL-NILs. The qRT-PCR gene expression data were also analyzed with principal component analysis. The Euclidean distance values were calculated from the scores of the first two principal components. The empirical gene expression network was constructed based on the Euclidean distance matrix. Figure 5 displays the network of the genes by expression analysis. The genes connecting in the network were potentially closely interacting genes in the signaling pathways. The network analysis showed that a RBO gene *zma100101532* was potentially involved with the expression of seven CDPK genes. Two WRKY factors *zma100383070* and WRKY83 were connected in the expression of a CDPK gene, *zma10365441*. All the CaM/CML genes appeared not to be connected (isolates) in the network.

## Discussion

The maize signaling pathway genes involve the activation of downstream proteins that lead to the defense responses in plant immune system. Upon the detection of the fungal PAMP pattern, an influx of calcium ions into the cytosol can be a result of activation of CNGCs by receptor kinase-bound nucleotidyl cyclases (Ma et al., 2009; Fountain et al., 2015a). These PTI responses increase cAMP or cGMP signaling molecules. The influx of Ca^2+^ ions activate the CDPKs which in turn active NADPH oxidase. CDPKs constitute a group of protein kinases with activity within plant extracts (Knight and Knight, 2001). These kinases along with calmodulin and calcium-regulated phosphatases are referred to as calcium sensors. These enzymes hold several roles within plant biology systems. CDPKs exhibit activation and function associated with hormone-related signaling, pathogen attack, and signaling during periods of environmental stress (Chen et al., 2004; Ludwig et al., 2004; Fountain et al., 2015a). CDPKs can also exhibit extensive cross-talk among differing signaling pathways in response to these stimuli (Ludwig et al., 2004). Specific CDPKs such as *ZmCDPK10* have previously been described to become activated significantly upon elicitation by fungal effectors (Murillo et al., 2001). In this study, seven CDPKs displayed a statistically significant difference among the maize QTL-NILs. The elucidation of signaling gene expression patterns of CDPKs associated with the resistance QTL-NILs can provide an avenue to the understanding of which genes are involved with PTI response signaling pathways.

Calmodulin/calmodulin-like proteins represent a wide array of functions within the plant. CaM/CMLs are calcium sensors and contribute to functions pertaining to metabolism, kinases, and phosphatases (Perochon et al., 2011). Previous studies have indicated that these proteins play pivotal roles in expression of plant immune responses (Perochon et al., 2011). However, analysis of the gene expression of three CaM/CML proteins in this study didn’t show significant differential expression. Furthermore, these CaM/CML genes (*zma100193164, zma100282040*, and *zma100285141*) appeared to be isolates and were not connected any other genes in the empirical gene expression network (Figure 5). Given the findings of a maize CDPK gene, *zma103653932*, in this study has both a CDPK domain and a CaM/CML domain in one gene, it represents a point for future analysis pertaining to the roles of such 2 in 1 genes (carrying both CDPK and CaM/CML functions in one gene) in potential plant defense against *Aspergillus flavus* infection.

NADPH oxidase converts O_2_ to the superoxide anion (O_2_). Eventually, the superoxide dismutase (SOD) converts O${}_{2}^{-}$ to hydrogen peroxide (H_2_O_2_). This molecule functions in cytosolic defense signaling (Fountain et al., 2015a). Respiratory burst oxidases or NADPH oxidases constitute an essential enzyme involved with ROS production and signaling. Several studies have been conducted to continue the knowledge of these enzymes. Proels et al. (2010) developed transgenic barley plants with knock down expression of a RBO homologous NADPH oxidase gene called HvRBOHF2. The results indicated that these knock-down plants exhibited increased susceptibility to fungal invasion of leaf segments. RBO genes have been implicated with the model organism *Arabidopsis* (Torres et al., 2002). Two genes were under investigation for their roles in ROS production during the defense response. These results concluded that *ArtbohD* and *ArtbohF* are required for ROS (reactive oxygen intermediate) production in *Arabidopsis*. These results provide evidence for effectiveness of ROS production in plant cell defenses. However, a delicate balance of ROS production must be maintained to prevent fungal invasion as well. The accumulation of excess amounts of H_2_O_2_ can lead to detrimental results to the plants as well. In response, the plant has the ability to efficiently remove excess H_2_O_2_ and thereby alleviate oxidative stress involved with damage to the plant. These processes can be accomplished by the activity of catalase enzymes. The *A. flavus* resistant lines Mp313E and Mp420 have been determined to exhibit lower steady-state levels of H_2_O_2_ than susceptible lines (Magbanua et al., 2007). Upon analysis of this research, one RBO, *zma10010532* provided a significant difference among the maize QTL-NILs. NIL-QTL4 displayed a significantly high expression of this RBO enzyme. The processes by which plant–fungus interaction affect ROS presence is a complex dynamic (Segal and Wilson, 2018). Further analysis could point to the direction of susceptibility of the plants versus the amount of ROS present as well as the neutralization of the ROS balance.

The production of ROS is the eventual result of fungal PAMP recognition signaling. As previously stated, the hypersensitive response and cell wall reinforcement are the conclusion following ROS production. The RBO ROS production pathway is a tightly regulated signaling mechanism working in conjunction with calcium signaling and phosphorylation events (Dietz et al., 2016). These signaling pathways will provide fundamental insights to *A. flavus* initiated signaling in experimental maize crops.

Another heavily studied topic of plant immune defense strategies involves the complex network of WRKY transcription factors. WRKY transcription factors can be best described by a highly conserved 60 amino acid sequence region. This conserved region is characterized as displaying a WRKYGQK peptide sequence at the terminal end of the protein and a novel zinc-finger DNA binding motif at the C terminal end. Furthermore, this conserved region will preferentially bind to a *W* box with the specific sequence TTGAC(C/T) (Eulgem et al., 2000; Rushton et al., 2010). WRKY transcription factors have three distinct groups to which they are classified based on the designated numbers of WRKY domains present and the structural characteristics of the zinc-finger motif) (Eulgem et al., 2000; Rushton et al., 2010). WRKY transcription factors play essential roles in biotic and abiotic stresses of the plant innate immune system. The activity of these factors can be described as both positive and negative regulators of these signaling pathways (Pandey and Somssich, 2009; Rushton et al., 2010). Several plant species have shown to require WRKY transcription factors in response to abiotic and biotic stresses and include *Arabidopsis* (Deslandes et al., 2002; Imran et al., 2018), rice (Qiu et al., 2007; Xie et al., 2017), cotton (Zhou et al., 2015) and tomato (Li and Luan, 2014; Liu et al., 2014; Karkute et al., 2018). The maize ZmWRKY33 has been reported to be induced and potentially utilized in the abscisic acid defense signaling pathway (Li et al., 2013). ZmWRKY19, ZmWRKY53, and ZmWRKY67 have been reported to exhibit higher expression levels in a resistant maize line, TZAR101 potentially via salicylic acid and ethylene defense signaling pathways (Fountain et al., 2015b). Of the 55 genes analyzed in this study, WRKY52, WRKY71, and WRKY83 displayed differential gene expression with a *p*-value significant at 0.05 level among the maize QTL-NILs. Further analysis of these maize WRKY transcription factors will provide the elucidation of complete signaling pathways involved with maize resistance to *A. flavus*.

## Data Availability Statement

All datasets generated for this study are included in the article/Supplementary Material.

## Author Contributions

XS and WW conceived and designed the experiments. FP, WW, GW, and XS performed the experiments. FP and XS analyzed the data and wrote the manuscript. WW and GW contributed and edited the manuscript. All authors have reviewed and approved the final manuscript.

## Conflict of Interest

The authors declare that the research was conducted in the absence of any commercial or financial relationships that could be construed as a potential conflict of interest.

## Footnotes

1 https://www.genome.jp/kegg
