# Supplementary material for: Differential Expression of Signaling Pathway Genes Associated With Aflatoxin Reduction Quantitative Trait Loci in Maize (Zea mays L.)
Source: Front Microbiol. 2019 Nov 26;10:2683. doi: 10.3389/fmicb.2019.02683 (PMC6901933; doi:10.3389/fmicb.2019.02683)
Supplement: Supplementary file 1 [file Table_1.docx]

**Supplemental Table 1. Summary of signaling pathway genes selected for phylogenetic tree and expression analyses**

| Genes | KEGG ID # | Maize GDB ID # | Primers | PCR Efficiency |
| --- | --- | --- | --- | --- |
| CDPKs |  |  |  |  |
|  | zma103654421 | GRMZM2G332660 | F_GCTGGACAAGTACGTGCAGA  R_CAGCATCCTGATCTCCGACT | 0.95 |
|  | zma103633012 | GRMZM2G080871 | F_GCATCAGAAGACAACGCAAA  R_AGGGCTTGCTAGTTGACGAA | 0.26 |
|  | zma1036647581 | GRMZM2G158721 | F_ GCGTCAGAAGACAATGCAAA  R_TTGCTGTTTGACGGAGTCTG | N/A |
|  | zma103641008 | GRMZM2G003059 | F_TGGAATGAACCTGACCATCA  R_CTGGACCTCACTGCTTCTCC | 0.77 |
|  | zma103625979 | GRMZM2G076634 | F_GGCAACGGTTACCTGGACTA  R_CGAAGAAGAGAAACGCCTTG | 0.42 |
|  | zma100192661 | GRMZM2G311220 | F_ AAGGACGGCAAGATCAGCTA  R_ATGCTGAGGCTGTTGAACCT | 0.91 |
|  | zma100273840 | GRMZM2G030673 |  |  |
|  | zma100381434 | GRMZM2G088361 |  |  |
|  | zma100281687 | GRMZM2G104125 | F_GAAATCGTAGGCAGCCCATA  R_AGTTTCTGCCCAAAATGGTG | 0.99 |
|  | zma103644628 | GRMZM2G006404 |  |  |
|  | zma100280693 | GRMZM2G099425 | F_CATGGCCCCAGAGGTACTAA  R_AGAATGGAGGGACACCACAG | 0.93 |
|  | zma103636213 | GRMZM2G047479 |  |  |
|  | zma100285166 | GRMZM2G028086 |  |  |
|  | zma100277352 | GRMZM2G040743 | F_GCTTCAAAAGGCTTCAGTGG  R_CTGTCGCTCTGCATCATTGT | 0.76 |
|  | zma100384477 | GRMZM2G347047 | F_AGCTTGCTGCATCTTCACCT  R_CACTACTCTTGCCCGACCAT | N/A |
|  | zma103653932 | GRMZM2G081310 | F_AAACCAAACGTTCCTGTTGC  R_TGAGCCAGCTTGAAATTCCT | 0.99 |
|  | zma542224 | GRMZM2G314396 |  |  |
|  | zma100384476 | GRMZM2G321239 |  |  |
|  | zma542227 | GRMZM2G441511 |  |  |
|  | zma606407 | GRMZM2G047486 | F_AGCTTGCTGCATCTTCACCT  R_CACTACTCTTGCCCGACCAT | 0.87 |
|  | zma103640839 | GRMZM2G347226 |  |  |
|  | zma100381559 | GRMZM2G463464 | F_GAGGAACAGCTTGCATGTGA  R_GGATGAGGCACACGGTAAGT | 0.98 |
|  | zma541988 | GRMZM2G320506 | F_AAGTGGACCAGGACAACGAC  R_CTCCCTGAACCCGATACTGA | 0.99 |
|  | zma103648263 | GRMZM2G012326 |  |  |
|  | zma103625819 | GRMZM2G027351 | F_GCAAAGGATTTGGTCAGGAA  R_ACCGGCTTAGAACAGCAGAA | 0.92 |
|  | zma103644148 | GRMZM2G121228 | F_AAGCAACCTAGGAGCGTGAA  R_AATTACACGGTGGCCTCAAG | 0.89 |
|  | zma103648992 | GRMZM2G365815 | F_TCAATCACCCATGGATCAAA  R_GGATAGGCACCCAGCTATGA | 0.52 |
|  | zma100286149 | GRMZM2G025387 | F_CTGACCCTTGGCCTTCAATA  R_AATCCATGGGTGGTTCAAAA | 0.98 |
|  | zma100383602 | GRMZM5G856738 |  |  |
|  | zma100285607 | GRMZM2G112057 | F_CTGGGAAGAAACGCAGAAAG  R_ATCCTTCGCGCTTTCAGATA | 0.73 |
|  | zma542526 | GRMZM2G154489 |  |  |
|  | zma54226 | GRMZM2G168706 | F_GTAGTCCCACCTGTGCCTGT  R_AGGTATGTCACCCCGAACTG | 0.53 |
|  | zma100381527 | GRMZM2G422576 | F_1TCCATCGTCGTTGTTGTTGT  R_AATCTTACGGCCCTTCCAGT | 0.99 |
|  | zma100217278 | GRMZM2G157068 |  |  |
|  | zma100284681 | GRMZM2G365035 | F_CCGTGACATTGTTGGAAGTG  R_GTCGTCTTCCACACAGCAAA | N/A |
| CaM-CaML | zma100273665 | GRMZM2G069922 |  |  |
|  | zma100280890 | GRMZM2G357595 | F_CTTCATTGAATGCTGCCTCA  R_ATGAAGGCCTCGTAGGTGAA | 0.70 |
|  | zma100282374 | GRMZM2G097827 | F_CGCGTACATCTTCTTCGACA  R_CCGTTCCTGTTCAGATCCAT | 0.95 |
|  | zma100193836 | GRMZM2G006790 |  |  |
|  | zma100193164 | GRMZM2G142693 | F_AGTTACACCGGGTCAGATGC  R_CAGAAGCAGCAAACAACCAA | 0.95 |
|  | zma100282040 | GRMZM2G155822 | F_ACTGGGACAAGAATGGAACG  R_GAATCTCCTTCAGCCGACAC | 0.96 |
|  | zma103647156 | ---- | F_GGACGAGAATGCTCAGAAGC  R_AGTGCGGCCAGTAGAAGAAA |  |
|  | zma103652986 | ---- |  |  |
|  | zma103655062 | GRMZM2G157241 |  |  |
|  | zma100281015 | GRMZM2G033846 | F_TGAGAGATCCATGGGCTGTT  R_AACCCCAGTCAACCACAGAC | N/A |
|  | zma100284831 | GRMZM2G088819 | F_GGTCGCGTTTGTTCAGAAAT  R_TTTCCATTGTGTGGGTGAGA | 0.91 |
|  | zma103647860 | ---- |  |  |
|  | zma100286265 | umc2252 |  |  |
|  | zma100283599 | GRMZM2G467184 |  |  |
|  | zma100282485 | GRMZM2G096271 |  |  |
|  | zma100381981 | GRMZM2G031329 |  |  |
|  | zma100283718 | GRMZM2G349655 |  |  |
|  | zma100273276 | GRMZM2G309327 |  |  |
|  | zma100382070 | GRMZM2G312661 |  |  |
|  | zma103650717 | GRMZM2G426046 | F_GGGTCGCAAGCTTATAGCAG  R_CGATACTGCGATGGATGATG | N/A |
|  | zma103652196 | GRMZM2G104523 |  |  |
|  | zma103636275 | GRMZM2G340313 |  |  |
|  | zma100282259 | GRMZM2G407626 |  |  |
|  | zma103651170 | GRMZM2G156377 |  |  |
|  | zma100280813 | GRMZM2G062673 |  |  |
|  | zma100285448 | GRMZM2G133588 |  |  |
|  | zma100276784 | GRMZM2G314292 |  |  |
|  | zma100277478 | GRMZM2G419452 |  |  |
|  | zma100284548 | GRMZM2G097900 |  |  |
|  | zma101027109 | ---- |  |  |
|  | zma103649813 | ---- |  |  |
|  | zma100286243 | GRMZM2G106945 |  |  |
|  | zma100280482 | GRMZM2G474755 |  |  |
|  | zma100284346 | GRMZM2G340807 |  |  |
|  | zma100857006 | ---- |  |  |
|  | zma100286292 | GRMZM2G152432 |  |  |
|  | zma100280614 | GRMZM2G847466 |  |  |
|  | zma100381663 | GRMZM2G072052 |  |  |
|  | zma103655921 | ---- |  |  |
|  | zma100284444 | GRMZM2G048846 |  |  |
|  | zma100283515 | GRMZM2G096228 |  |  |
|  | zma103645035 | ---- |  |  |
|  | zma103635808 | GRMZM2G005557 |  |  |
|  | zma103637586 | GRMZM2G355525 |  |  |
|  | zma100272455 | GRMZM2G324643 |  |  |
|  | zma606468 | GRMZM2G149923 |  |  |
|  | zma100285141 | GRMZM2G115628 | F_CAAGACGAAGGAACCAGGAG  R_GGCAGAACGAGTCGAGTTTC | 0.90 |
|  | zma100285551 | GRMZM2G146720 |  |  |
|  | zma1002502442 | ---- |  |  |
|  | zma100281919 | ---- |  |  |
|  | zma100381315 | GRMZM2G430600 |  |  |
|  | zma542420 | GRMZM2G152891 |  |  |
|  | zma100217114 | GRMZM2G39244 |  |  |
|  | zma103634668 | GRMZM2G044963 |  |  |
|  | zma103639205 | GRMZM2G029107 |  |  |
|  | zma541661 | GRMZM2G004703 |  |  |
|  | zma542415 | GRMZM2G067511 |  |  |
|  | zma542415 | GRMZM2G117582 |  |  |
|  | zma542465 | ---- |  |  |
| RBO | zma100274717 | GRMZM2G089285 | F_TGGTACTCGCACCATGTGTT  R_CGACATACATCCACGTCGTC |  |
|  | zma103643238 | GRMZM2G448185 | F_CAGCAGGGACAAGAAGAAGG  R_TAGACGACGATGAGCAGGTG | 0.92 |
|  | zma100037794 | GRMZM2G138152 |  |  |
|  | zma100101532 | GRMZM2G043435 | F_GAGCTTTGGCAACTGGAGAC  R_CCAGCAAGGTTCTGGCTTAG | 0.97 |
|  | zma100136880 | GRMZM2G441541, rbohD |  |  |
|  | zma103648044 | GRMZM2G037993 |  |  |
|  | zma103648040 | GRMZM2G065144 | F_TGTTCTGGGACCAAATCTCC  R_ATCTCCTCCTCGGTGATCCT | 0.77 |
|  | zma103648043 | GRMZM2G358619 |  |  |
|  | zma100381459 | GRMZM2G300965 | F_CTCATCAGCATCGTCAAGGA  R_CGCGTGCACCAGTAGAAGTA | N/A |
|  | zma103635158 | GRMZM2G022547 | F_ACCGTGACAGCCTTGGATAC  R_TTTCGAGCTATGCGTTGTTG | 0.76 |
|  | zma100384248 | GRMZM2G034896, IDP491 |  |  |
| WRKY | zma100275623 | GRMZM2G151407 | F_CGCCCTTTCTAGTTCAGTCG  R_GAGACTTCTTGGGCTTGGTG | 0.95 |
|  | zma100383070 | GRMZM5G816457 | F_TCTTCTCTCTCCGGCGTAAA  R_GGGGAGTCAGGAAAAGAAGG | 0.94 |
|  | zma100384128 | GRMZM2G052671 | F_CCTCCACCACCAATACCATC  R_ATACTTGCGCCACCGATAAC | 0.92 |
|  | zma103641750 | GRMZM2G031963 | F_CTCCAGTCACAGGTTGAGCA  R_CAGCTCTGGATTGGAAGACC | N/A |
|  | zma103654474 | GRMZM2G027972 | F_CGTGAATCAGAAACCGACCT  R_CACCAGATGGAATTGTCACG | N/A |
|  | WRKY 83 | GRMZM2G012724 | F_ACCTGATCGCGTCTCAGTCT  R_CCTTGAAGGAAGGGAAGGAC | 0.99 |
