# Supplementary material for: Differential Expression of Signaling Pathway Genes Associated With Aflatoxin Reduction Quantitative Trait Loci in Maize (Zea mays L.)
Source: Front Microbiol. 2019 Nov 26;10:2683. doi: 10.3389/fmicb.2019.02683 (PMC6901933; doi:10.3389/fmicb.2019.02683)
Supplement: Supplementary file 2 [file Table_2.docx]

| **Supplemental Table 2: Maize genes obtained through BLAST of Arabidopsis WRKY Transcription Factors** | | | | | |
| --- | --- | --- | --- | --- | --- |
| Gene type (Arabidopsis) | KEGG ID # | Maize Orthologue | Maize GDB ID # | Primers | Primer Efficiency |
|  |  |  |  |  |  |
| **Arabidopsis** |  | **Maize** |  |  |  |
| WRKY 29 | AT4G23550 | WRKY 60 | GRMZM2G383594 | F_CTTCCACTCGTTCCAGCTTC  R_TCTCTTGACTGCCGAGGAAT | N/A |
| WRKY 29 | AT4G23550 | WRKY 13 | GRMZM2G156529 | F_GTAGCAGCAACAACGACTGG  R_CACATCGTCCTTCTGTGGTG | N/A |
| WRKY 29 | AT4G23550 | WRKY 49 | GRMZM2G006497 | F_GACCTTTCTCACGCCTTACG  R_TTTCTGACACAGGGTGCTTG | 0.73 |
| WRKY 25 | AT2G30250 | WRKY 83 | GRMZM2G012724 | F_ACCTGATCGCGTCTCAGTCT  R_CCTTGAAGGAAGGGAAGGAC | 0.99 |
| WRKY 25 | AT2G30250 | WRKY 43 | GRMZM2G148087 | F_CAAAGCAGTTCCCACCTCAT  R_ACCGATCGTGTGTGTGTGTT | 0.76 |
| WRKY 25 | AT2G30250 | WRKY 58 | GRMZM2G076657 | F_GCATGCAGAAGATGGTCAGA  R_ATGCTGAAGAGTTGCCGTCT | 0.8 |
| WRKY 22 | AT4G01250 | WRKY 8 | GRMZM2G038158 | F_AGAGATGTGCCGTCTGTCCT  R_AGGGCTGACTACCGATGCTA | 0.7 |
| WRKY 22 | AT4G01250 | WRKY 2 | GRMZM2G048450 | F_CTGGTGGAGGACATGGAGAT  R_GCCAAGGGGAGTTTAGGAAG | 0.68 |
